# Dragon Fruit Foliage: An Agricultural Cellulosic Source to Extract Cellulose Nanomaterials

**DOI:** 10.3390/molecules26247701

**Published:** 2021-12-20

**Authors:** Tuyet Phung Thi Anh, Toan Viet Nguyen, Phuong Thi Hoang, Phuong Vu Thi, Thoa Nguyen Kim, Quyen Nguyen Van, Chien Nguyen Van, Yen Dao Hai

**Affiliations:** 1 Institute of Chemistry, Vietnam Academy of Science and Technology, 18 Hoang Quoc Viet, Cau Giay, Hanoi 11307, Vietnam; phungthianhtuyet98@gmail.com (T.P.T.A.); Viettoan1997na@gmail.com (T.V.N.); hoangphuong15@gmail.com (P.T.H.); vuphuong19041999@gmail.com (P.V.T.); 2 Institute of Biotechnology, Vietnam Academy of Science and Technology, 18 Hoang Quoc Viet, Cau Giay, Hanoi 11307, Vietnam; nkthoa.ibt@gmail.com; 3 Department of Advanced Materials Science and Nanotechnology, Vietnam Academy of Science and Technology, University of Science and Technology of Hanoi (USTH), 18 Hoang Quoc Viet, Cau Giay, Hanoi 11307, Vietnam; 4 Institute for Tropical Technology, Vietnam Academy of Science and Technology, 18 Hoang Quoc Viet, Cau Giay, Hanoi 11307, Vietnam; chiennguyen@itt.vast.vn

**Keywords:** cellulose nanomaterial, nanocellulose adsorbent, agricultural cellulosic material

## Abstract

In this report, we focus our effort to extract cellulose nanomaterials (CNs) from an agricultural cellulosic waste, Dragon Fruit foliage (DFF). DFF was first pretreated by several mechanical treatments and then bleached by chemical treatment to obtain bleached DFF. CNs were then produced from the hydrolysis of the bleached DFF catalyzed by sulfuric acid. We obtained CNs with a small diameter (50 to 130 nm) and length (100 to 500 nm) and a height of 3 to 10 nm. The CNs have a high crystallinity (crystallinity index 84.8%), high −COOH content (0.74 mmol·g^−1^), good thermal stability and a good Cu (II) adsorption capacity with an adsorption maximum of ~103 mg·g^−1^. These findings demonstrated the great potential of converting many agricultural cellulosic wastes into valuable cellulose nanomaterials.

## 1. Introduction

Biodegradable, renewable and sustainable nanomaterials play an essential role in solving not only technological challenges but also social impacts. Of many, cellulose nanomaterials (CNs) have recently emerged as a promising biomaterial due to their possible applications, such as in biomedical devices, [1,2,3] water purification, [1,4,5] energy storage, [6] tissue engineering [7,8] and packaging [9,10].

CNs, (C_6_H_5_O_6_)_n_ chemical formula, hold a variety of unique properties, for example, high mechanical strength, biodegradable, surface tunable chemistry and reinforcing abilities [1,11,12,13,14]. Xia et al. recently reported a cost-effective bioplastic based on nanocellulose exhibiting a high tensile strength (12 MPa), water stability and improved thermal stability, and more importantly, the bioplastic can be biodegraded by microorganisms in the natural environment. Liu et al. reported that the maximum adsorption capacity of Cu (II) on modified CNs is 73 mg·g^−1^, and importantly, the adsorption of Cu (II) ions onto modified CNs is extremely fast, less than 1 min, and monolayer adsorbed Cu (II) ions could form on the surface of CNs [15]. Aji P. Mathew et al. later reported the use of nanocellulose as a platform for the formation of metal oxide nanoparticles, Cu_2_O, on its surface [16,17]. Interestingly, the nanoparticle can be spontaneously grown on the carboxylated CNs by the adsorption of Cu (II) ions from water and these hybrid nanomaterials can not only remove dye waste but also be used as antimicrobial materials.

CNs are now produced at a pilot ton-per-day scale by the hydrolysis of cellulosic biomaterial [18]. The cellulosic sources for the production of CNs are usually cotton, wood, hemp, flax and other non-woody resources, such as agricultural residues, fruit and vegetable waste and grasses [1,18,19]. However, there are still various cheap cellulosic materials available in nature and especially in an unexplored agricultural industry.

**Figure 1:**
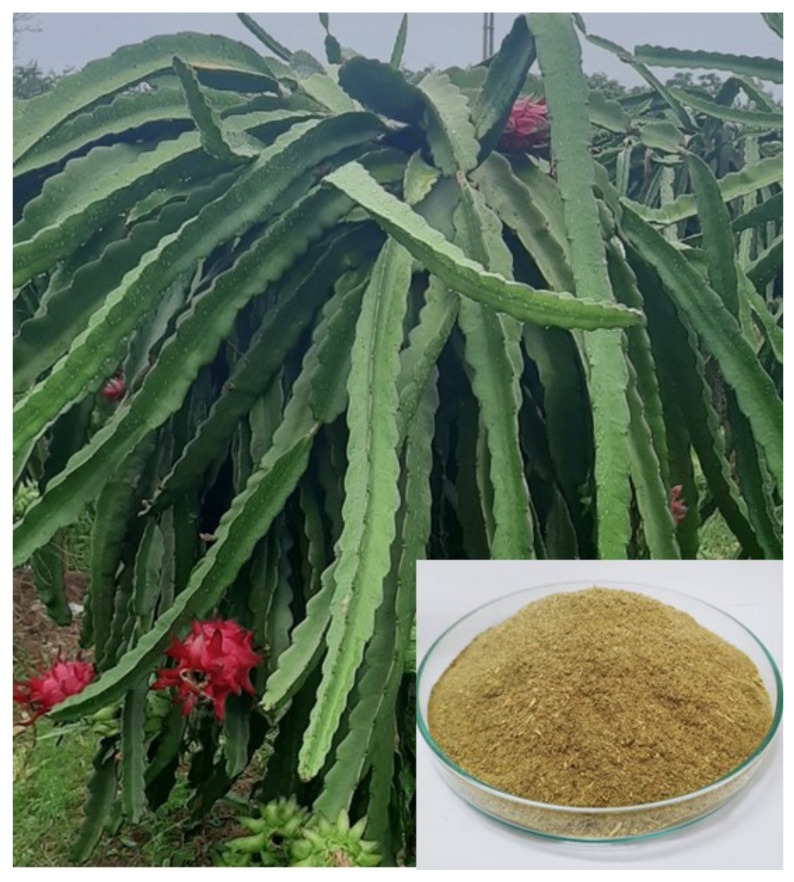
Dragon Fruit foliage (DFF) during the harvest season in Hai Duong, Vietnam. Collected DFF were air dried and mechanically milled into fine particles, as shown in the inset.

Dragon Fruit or pitaya, as pictured in Figure 1, is native to tropical regions of North, Central and South America. It was brought to Vietnam by the French in the 19th century and then it was commercialized. Nowadays, it has been widely planted in provinces and cities nationwide with more than 55,000 hectares. Dragon Fruit foliage (DFF) is considered one of the agricultural by-products obtained by pruning Dragon Fruit trees and is mostly treated as agricultural waste in Vietnam. DFF is fast growing, inexpensive, sustainable and has a high rate of reproduction. This makes DFF an interesting candidate for the production of CNs.

In the present study, we selected dragon fruit foliage as a raw cellulosic material to produce cellulose nanomaterial by the hydrolysis process catalyzed by sulfuric acid. The main chemical components of DFF, such as cellulose, hemicellulose and lignin, will be first quantified and then the production of CNs will be studied and the physiochemical properties of CNs will be characterized by several physical techniques. We demonstrated that CNs can be produced in high yield (80%), and these CNs can be used as a promising adsorbent to remove metal ions, for example Cu (II) ions.

## 2. Materials and Methods

### 2.1. Preparation of Raw Material and Chemicals

Dragon Fruit foliage (DFF) was air-dried for a week prior to use and mechanically milled into fine dried practices, as shown in Figure 1, stored in a sealed container. Analytical grade chemicals, including sodium hydroxide, hydrogen peroxide, acetic acid and sulfuric acid, were purchased from Sigma-Aldrich and used without further purification.

### 2.2. Chemical Composition Analysis

Extractive and ash content: The procedure was adapted from Tappi T 211 om-02. The DFF was then extracted with a mixture of ethanol/toluene (9:1 and 1:1 in *v/v*, respectively) in the Soxhlet apparatus for 6 h each time and 3 times for each mixture. The DFF was washed with ethanol and then dried in an oven under vacuum overnight. The dewaxed sample was first charred for 3 h and then transferred into the oven heated at 575 °C for 6 h. The ash was removed and then determined after cooling down to an ambient temperature under vacuum.

Lignin Content: The lignin content of dried DFF was estimated based on the technical report given by the national renewable energy laboratory (NREL/TP-510-42618). In short, the dried DFF sample was first treated with 72% H_2_SO_4_ (with ratio 1/15 *w/v*) at 30 °C for 2 h. The solution was diluted with 300–400 mL of water and then transferred to an autoclave at 120 °C for 1 h. The lignin content in the filtrated solution was then quantified by Ultra violet spectroscopy at 205 nm using an absorptivity value of 110 L.g^−1^.cm^−1^ to determine the acid soluble lignin content (ASL).

The filtered solids were washed with hot deionized water and then dried at 105 °C overnight to determine acid-insoluble lignin (AIL). The total lignin was estimated using the formula below:

%Total Lignin = %ASL + %AIL

Hemicellulose Content: The sample after pretreated Soxhlet was extracted with 10% KOH at 25 °C for 16 h with a solid-to-liquid ratio of 1:25 g/L. The hemicellulosic fraction (Ha) was precipitated by neutralizing the filtrate with an HOAc of pH 5.5 to 6.0. Next, the filtrate was concentrated under reduced pressure, and pure ethanol was added to the filtrate with continuous stirring to a final concentration of 10% (*v/v*). The precipitated hemicellulosic polymer was recovered by centrifugation and marked as H10. The supernatant was further sequentially fractionated by graded precipitation in ethanol concentrations of 20%, 35%, 50%, 65% and 80%, leading to hemicellulosic fractions H20, H35, H50, H65 and H80, respectively. All hemicellulosic fractions were dialyzed against distilled water, freeze-dried and then the hemicellulose content was determined.

### 2.3. Bleached DFF

The extracted DFF samples were soaked with 3% NaOH (1:10, *w/v*) at 120 °C for 15 min in an autoclave, then filtered and washed with hot distilled water several times. This step was repeated until the filtrated solution remained colorless. Afterward, the remaining solid was neutralized by washing with distilled water until pH ~7. The sample was then air dried overnight and stored for the next steps. The DFF sample was bleached in an aqueous solution containing H_2_O_2_ (1.3%, *w/w*) and acetic acid (0.1%, *v/v*) (this solution was prepared by combining 74 mL 35% *w/w* H_2_O_2_ in H_2_O with 2 L H_2_O and adding 2 mL of glacial acetic acid) by stirring at 70 °C for 2 h. The solids were filtered off and washed with H_2_O (3 × 500 mL). This bleaching/washing cycle was repeated once. After two cycles, the solids were washed with H_2_O until the pH was neutral and dried for 24 h at ambient temperature and pressure.

### 2.4. Isolation of Cellulose Nanomaterials

Cellulose nanomaterials (CNs) were produced from the hydrolysis of the bleached cellulose derived from DFF samples using sulfuric acid. The bleached cellulose was hydrolyzed using sulfuric acid solution (64%) with the ratio of 1:15 at 45 °C for 180 min. To enhance the dispersion of bleached cellulose, the suspension was sonicated by using a Elmasonic S 300 H for 20 min. After that, cooled distilled water was added to quench the hydrolysis reaction. The solution was then washed with H_2_O several times by centrifugation at 10,000 rpm for 15 min. The white mixture was subjected to a dialysis step against distilled water until reaching a neutral pH. The yield of extracting CNs was calculated as approximately 80%. The CNs were then homogenized for 5 min in an ice bath by a high intensity ultrasonic processor (UP200St, Hielscher).

### 2.5. Characterization

Fourier transform infrared (FT/IR 4700, JASCO, Tokyo, Japan) equipped with the attenuated total reflectance (ATR) accessory was used to analyze the chemical structure of the samples. The samples were dried and taken at a wavenumber range of 400 to 4000 cm^−1^ with a resolution of 4 cm^−1^ and 16 scans. Thermogravimetric analysis (TGA, Setaram) was used to analyze the thermal stability and degradation behavior of CNs, DFF and bleached cellulose. The analysis was performed between ambient and 700 °C under air, with a flow rate of 2.5 L/min at a heating rate of 10 °C/min.

X-ray Diffraction. XRD patterns of the fibers at different stages were measured by using a D8 ADVANCE X-ray powder diffractometer (Bruker, Germany), operated at 40 kV and 30 mA using Ni-filtered Cu K radiation (λ = 0.15406 nm). The samples were scanned at the ambient condition over scattering 2 angles from 10° to 70° with a scanning rate of 0.03°/0.7 s. The crystallinity index (CrI) of each sample was calculated according to Segal’s method, as presented by the formula below.

$$\mathrm{CrI}\;\left(\%\right)=\frac{A_{200}}{A_{200}+A_{am}}\times 100$$

where *A*_200_ is the peak area at plane (200) characterized for the crystallinity domain, while *A_am_* is the peak area at the (101) plane characterized for amorphous domain in cellulose.

Particle Size and Zeta Potential Measurement: Particle size and zeta potential of the samples were measured using a Nano-ZS particle size and zeta potential analyzer (Malvern Instrument, Worcestershire, UK)). Suspensions of the CNs (0.05 wt%) were sonicated for 10 min using Vibra Cell^TM^ VCX750 Ultrasonic Processors at 325 W (ON/OFF, 30 s/15 s). Measurements were carried out three times for each sample.

Qualifying −COOH Contents: The −COOH content of the sample was determined by an electrical conductivity method [20,21] with Hach HQ40D Dual-Input Multiparameter Meter, adapted from the Canadian Standards Association (CSA), which has recently published a suggested protocol for the determination of physical and chemical properties of CNMs entitled Cellulosic Nanomaterials—Test Methods for Characterization (CSA Z5100-14). Freeze-dried samples (0.25 g) were suspended in 100 mL deionized water. Before titration, 250 μL 0.1% (*w/v*) NaCl was added to increase sample conductivity, and the pH was adjusted to 2.5–3 to protonate all carboxylate groups. Conductimetric titration was performed with 0.1 N NaOH as a titrant. The −COOH content was calculated using the following equation:

$$-\mathrm{COOH}\;\mathrm{content}=\frac{C\left(V_{1}-V_{2}\right)}{W}\;\left(\mathrm{mmol}/g\right)$$

where V_2_ and V_1_ are the volume of titrant required to neutralize the carboxylic groups, C is the NaOH concentration (mol/L) and W is the dry sample weight. Each sample was measured three times.

Atomic Force Microscopy (AFM): AFM, Asylum research MFP-3D origin, was used to image CNs. AFM was performed at an ambient temperature in tapping mode with a scan rate of 1 Hz using a tip from Budget sensor (AFM tap 300 G, resonance frequency 300 kHz, force constant 40 nN/m). AFM images were taken of 5 μm × 5 μm and then all data were analyzed by Gwyddion 2.59 software.

Cu (II) Adsorption: The Cu (II) adsorption capacity of all raw DFF, bleached DFF and CN samples was evaluated by immersion of 100 mg of CNs in 200 mL of the Cu (II) 1 g. L^−1^ salt solutions. The contact time was modified from 0.5 to 5 h and the Cu (II) concentration remaining in the solution was then monitored by ultraviolet spectroscopy. The experiments were done at pH ~5 and room temperature. The adsorption capacity of the CNs was calculated according to the following equation:

$$q_{e}=\;\frac{(C_{0}-C_{e})\;\times \;V}{m}$$

where q_*e*_ is the amount of pollutant adsorbed (mg·g^−1^), *C*_0_ (mg·L^−1^) and *C_e_* (mg·L^−1^) are the initial pollutant solution concentration and the equilibrium concentration of pollutant in the bulk solution. *V* (L) is the volume of solution, and m (g) is the weight of the adsorbent.

## 3. Results

### 3.1. Chemical Components

The chemical components of the DFF were qualified, and the results are shown in Table 1. Obviously, the DFF source can be considered as lignocellulosic fibers, which are composed of cellulose (~31%), lignin (~14%) and hemicellulose (~28%) components, extractives and ash components in DFF of 15% and 12%, respectively. The amount of cellulose is significantly high and comparable to other cellulosic sources, especially for agricultural cellulosic sources. We note here that the amount of cellulose could be improved by peeling off the outer fresh DFF; however, it could be impractical in industry.

**Figure 2:**
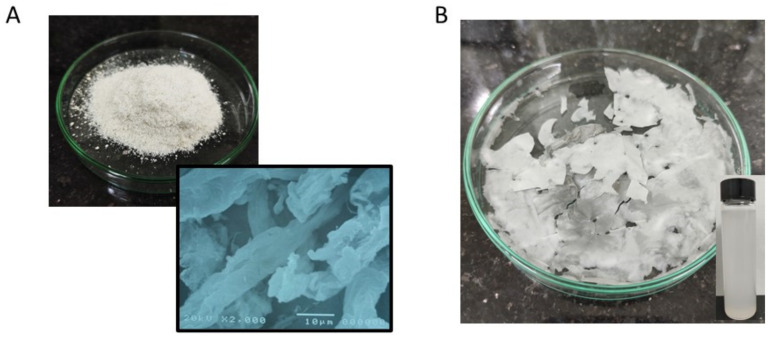
(**A**) Image of bleached DFF with scanning electron microscopy (SEM) images. (**B**) Image of dried cellulose nanomaterials, CNs, obtained after the freeze-drying process with an inset showing the dispersion of CNs in water after 7 days.

The raw DFF was then bleached as mentioned above to remove all hemicellulose, lignin, extractives and other inorganic minerals. The bleached DFF was then pictured as shown in Figure 2A. As revealed by SEM, the bleached DFF are microfibers with a diameter in the range of 5 to 20 μm with good homogeneity, which is consistent with other bleached cellulose originating from non-woody sources [25,26,33].

### 3.2. Chemical Structures

**Figure 3:**
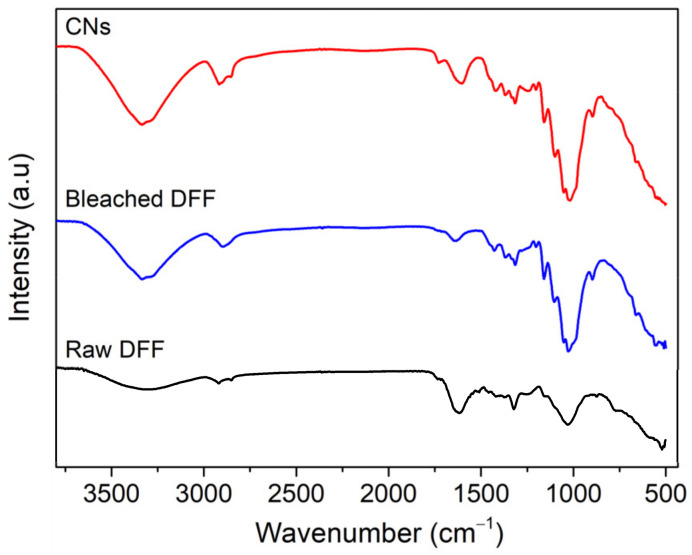
Fourier transform infrared spectra of raw DFF (**black**), bleached DFF (**blue**) and CNs (**red**).

The chemical structures of the CNs produced by hydrolysis using H_2_SO_4_ are compared to the raw DFF and bleached cellulose, as shown in Figure 3. As shown, in the spectra of raw DFF, there are vibrational bands at 1514 and 1460 cm^−1^ assigned to C = C and C = C-C groups vibration stretching from aromatic hydrocarbon of lignin [21,28,33]. These bands almost disappeared in the spectra of bleached cellulose and CNs, which indicates that the lignin was completely removed in bleached cellulose and CN samples. The vibrational band at 1242 cm^−1^, assigned to the COO- group, was observed for all samples, which could result from the bleaching process, and as shown later, these COO- groups enhance the adsorption capability of CNs. The band at 1641 cm^−1^ could be assigned to the vibration binding of water molecules adsorbed into cellulose. These bands at 1426 and 1315 cm^−1^ can be assigned to CH_2_ symmetric bending groups of cellulose and the intensity of these bands in bleached cellulose and CNs is much stronger than the raw DFF, which again indicates that the cellulose content in bleach cellulose and CNs is much greater than the raw DFF. In addition, we observed the vibrational bands between 899 and 1163 cm^−1^, which usually assigns to β-(1-4) glycosidic ether linkages and the β-glycosidic linkages, C-O-C glycosidic symmetric stretching, C-OH stretching vibration and C-O stretching, indicating the presence of cellulose structure. Overall, the FT-IR data indicated the removal of lignin and hemicellulose, while the cellulose structure was maintained after hydrolysis in H_2_SO_4_.

### 3.3. Crystallinity

**Figure 4:**
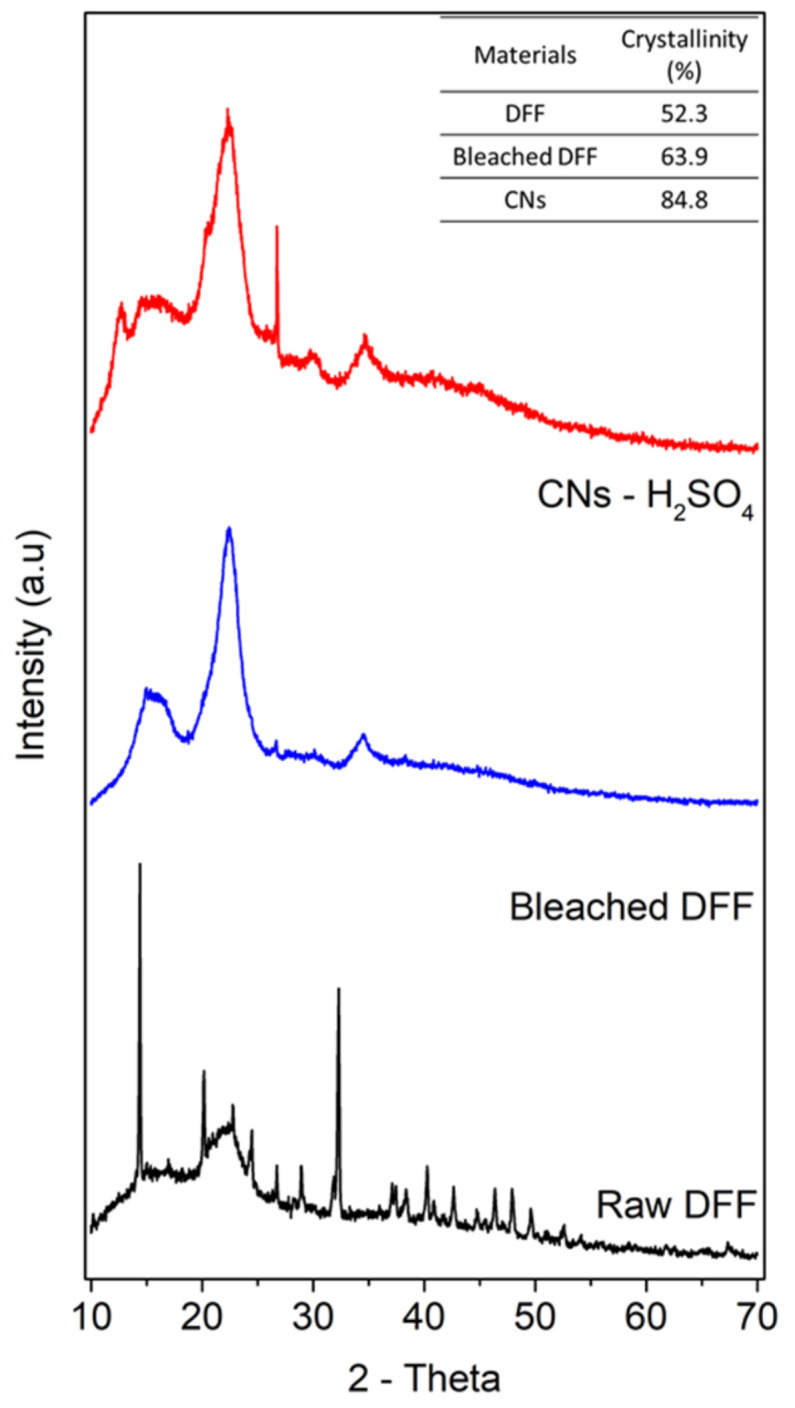
XRD patterns of a raw DFF (**black**), bleached DFF (**blue**) and CN (**red**) samples, an inset table showing the crystallinity index.

The crystalline structure and crystallinity of all raw DFF, bleached DFF and CN samples were determined by XRD analysis, and the result is presented in Figure 4. In general, al samples show the typical cellulose I structure, which is characterized by the main diffracted peaks at 14–17°; 22.3° and 34° corresponding to (110), (101), (200) and (400) planes [21,33]. The result is expected for natural lignocellulosic sources. It is important to note that in the spectra of raw DFF, there are diffraction planes at 2-theta of 15°, 25° and 37°, which could assign to the presence of calcium oxalate [34], a mineral existing in DFF materials, as shown in Figure 4. The XRD data also indicated that the inorganic mineral was completely removed while the cellulose structure remained after several chemical and physical treatments. We note here that in our experiments, low concentration alkaline solution (<5%) used to treat the raw DFF cannot transform cellulose I to cellulose II arising when high concentration alkaline is used. Another observation is the intensity of these peaks in bleached DFF, especially CNs, is greater than that of raw DFF, which suggests the removal of most amorphous non-cellulosic components, lignin and hemicellulose.

The crystallinity index (*CrI*) of all samples was estimated using Segal’s method described above and the results are shown in the inset in Figure 4. The *CrI* of the raw DFF was 52.5%, and the degree of bleached DFF was increased to 63.9%. The increase was expected to be caused by the removal of hemicellulose and lignin. Similar increases in *CrI* after chemical treatment have also been reported for other cellulosic materials, such as wood, bamboo, rice straw, water hyacinth, Doum tree and flax. The *CrI* of CN samples was approximately 84.8%, much greater than the raw DFF and bleached DFF samples, and its value is also consistent with the cellulose nanomaterial produced by the hydrolysis of other cellulosic sources catalyzed by H_2_SO_4_ [18]. The CNs showed the narrowest and sharpest pattern, especially at 2θ ~22° and an additional pattern peaked at 14° assigned to (101) planes. The higher *CrI* value of CNs compared to those of raw DFF and bleached DFF is caused by the hydrolysis of the amorphous regions of cellulose microfibers during the acid hydrolysis reaction. The high crystalline CNs in our study could be considered good nano-reinforcing agents for polymer nanocomposite application [1,35].

### 3.4. Thermal Stability of CNs

**Figure 5:**
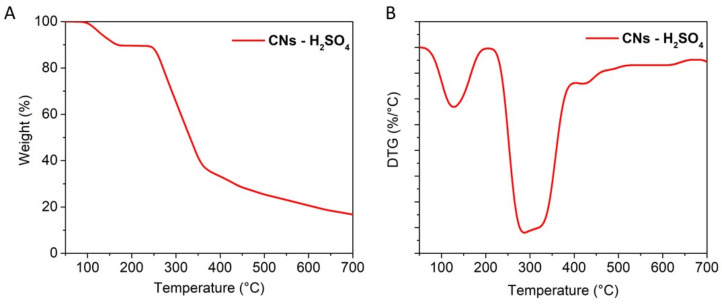
(**A**) Thermogravimetric Analysis TGA and (**B**) Differential Thermal Analysis (DTG) curves of CN samples recorded in air.

The thermal stability of CNs was evaluated by TGA/DTG analysis as presented in Figure 5. As expected, the decomposition of the CNs was occurred at 3 steps from low to moderate temperatures, as expected for lignocellulosic material. Figure 5 shows that the initial step of degradation occurred at T_onset_ ~110 °C, which could be assigned to the weight loss (10%) of the amount of moisture in CNs, which was enhanced due to its hydrophilic behavior after the hydrolysis process. The second step of the degradation was from 250 °C to 360 °C where the crystalline region started to decompose with a major weight loss (~60%) due to gas phase transition and tar formation. The T_onset_ of the second step (250 °C) is also consistent with many CNs generated from the hydrolysis of cellulosic material catalyzed by H_2_SO_4_ [36]. During the last stage from 360 °C to 600 °C, the crystalline structure was completely destroyed and the cellulose decomposed into D-glucopyranose monomeric units, then further decomposed into volatiles and tar [32,33].

### 3.5. Dimension and Surface of CNs

**Figure 6:**
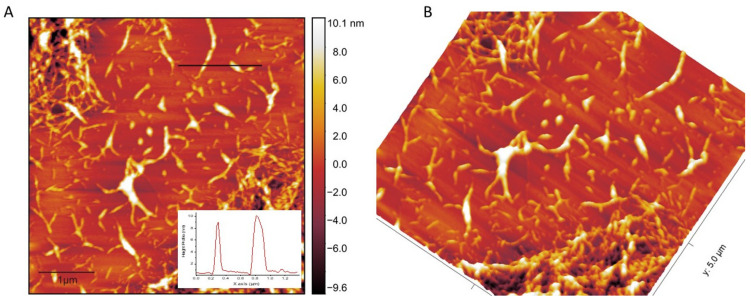
Atomic force microscopy of CNs (**A**) Z-height topography with height profile (**B**) 3D image collected from tapping mode.

Figure 2B shows the image of CNs produced from the hydrolysis of bleached DFF catalyzed by sulfuric acid in 2 h. The dimension of CNs was first investigated by atomic force microscopy in tapping mode. A droplet of diluted CN suspensions (~0.001 wt%) was first deposited on a fresh mica substrate (2 × 2 cm^2^), and the sample was then allowed to dry at ambient temperature in a vacuum. Figure 6 presents the AFM data of the CNs. Figure 6A displays the height topography collected from piezo, while Figure 6B presents the 3D topography converted from the same image in Figure 6A. The AFM data revealed that the CNs exhibited a diameter in the range of 50 to 130 nm, while the length of CNs was estimated in the range of 100 to 500 nm. The aspect ratio, length/width ratio, was calculated in range of 2 to 5 and its value is significantly smaller in comparison with other cellulosic sources [11,28,33]. As shown in an inset in Figure 6A, the height of CNs was estimated in the range of 3–10 nm, which is consistent with that of CNs produced from other sources [28,33,37]. Moreover, AFM data revealed that there is aggregation of CNs during the drying process, which is consistent with several other reports [18,38,39], and the aggregation phenomenon in the drying process has been studied and will be published elsewhere. Interestingly, the particle size of CNs in solution was estimated as 320 ± 20 nm, which is in good agreement with the result estimated from AFM data. The zeta potential measured from CNs dispersion was −60 mV, which is expected to be caused by −SO_3_^−^ and possible COO- functional groups, the low zeta potential caused a very good dispersion of CNs in water after 7 days, as shown in an inset in Figure 2B. We noticed here that −COOH contents on surface of CNs was estimated to be 0.74 mmol·g^−1^ which is surprisingly high and close to the value of CNs produced by TEMPO [40].

### 3.6. Cu(II) Adsorption

Herein, we attempted to utilize the CNs generated from DFF as an adsorbent to remove Cu (II) ions in water (pH ~4). We monitored the Cu (II) concentration with respect to time in the range of 0.5 to 5 h with a timing step of 0.5 h. We observed that the kinetics of the adsorption process took place quickly, almost completely after 0.5 h and the maximum adsorption was slightly changed after 0.5 h. We note here that due to the limitation of our setup, we are unable to perform the adsorption experiment in a smaller timing window. Our observation agreed well with the report given by Mathew et al. [15]. We also observed that the high maximum Cu(II) adsorption of CNs was 103 mg·g^−1^ which probably resulted from the high COOH concentration on the surface of CNs.

## 4. Conclusions

In summary, we reported that cellulose nanomaterials can be successfully isolated from dragon fruit foliage, an agricultural cellulosic waster in Vietnam. The obtained CNs were fully characterized by several physical techniques, such as atomic force microscopy, scanning electron microscopy and infrared spectroscopy. The CNs exhibited a relatively small diameter (50 to 130 nm) and length (100 to 500 nm) with a height of 3 to 10 nm. The CN materials show a good adsorption capacity toward Cu (II) ions in water, and the adsorption maximum is approximately 103 mg·g^−1^. These findings demonstrated a great opportunity to convert many agricultural cellulosic wastes into cellulose nanomaterials, which can be utilized as antimicrobial materials and heavy metal adsorbents.

## Figures and Tables

**Table 1:** Chemical composition of the studied DFF source and several cellulosic materials.

Sources	Cellulose	Hemicellulose	Lignin	Extractives	Ash	Refs.
Dragon Fruit foliage	30.8	27.5	14.4	15.4	11.8	This work
Pseudostem banana	34.5	25.6	12	8.9	13.9	[22]
Straw	33.5	27.1	25.8	-	2.5	[23,24]
Durian peel	60.45	13.09	15.45	-	-	[25]
Pineapple leaf	73.4	7.1	10.5	-	-	[26,27]
Water hyacinth	25	33	10	5.5	2.0	[28]
Corn (husk)	56.4	21.9	7.6	17.9	3.65	[29,30]
Corn (cob)	54.8	14.4	9.4	15.9	3.2	[31,32]
Doum tree	30.86	18.57	33.12	-	2.23	[33]

## Data Availability

Data are contained within the article.

## Sample Availability

Samples of the compounds are available from the authors.
